# Clinical Translatability of “Identified” Circulating miRNAs for Diagnosing Breast Cancer: Overview and Update

**DOI:** 10.3390/cancers11070901

**Published:** 2019-06-27

**Authors:** Anna Maria Grimaldi, Mariarosaria Incoronato

**Affiliations:** IRCCS SDN, 80143 Naples, Italy; agrimaldi@sdn-napoli.it

**Keywords:** miRNAs, circulating miRNAs, breast cancer

## Abstract

The effective management of patients with breast cancer (BC) depends on the early diagnosis of the disease. Currently, BC diagnosis is based on diagnostic imaging and biopsy, while the use of non-invasive circulating biomarkers for diagnosis remains an unmet need. Among the plethora of proposed non-invasive biomarkers, circulating microRNAs (miRNAs) have been considered promising diagnostic molecules because they are very stable in biological fluids and easily detectable. Although the discovery of miRNAs has opened a new avenue for their clinical application, the clinical translatability of these molecules remains unclear. This review analyses the role of circulating miRNAs as BC diagnostic biomarkers and focuses on two essential requirements to evaluate their clinical validity: i) Specificity and ii) consistent expression between the blood and tissue. These two issues were analyzed in depth using the Human miRNA Disease Database (HMDD v3.0) and the free search engine PubMed. One hundred and sixty three BC-associated miRNAs were selected and analyzed for their specificity among all human pathologies that shared deregulation (291) and consistent expression in the bloodstream and the tissue. In addition, we provide an overview of the current clinical trials examining miRNAs in BC. In conclusion, we highlight pitfalls in the translatability of circulating miRNAs into clinical practice due to the lack of specificity and a consistent expression pattern between the tissue and blood.

## 1. Introduction

Breast cancer (BC) is the most commonly diagnosed cancer (11.6% of the total cases) in females worldwide, with approximately 2.1 million new cases diagnosed in 2018, and the leading cause of cancer-related death in over 100 countries (mortality rate: 6.6%) [1]. Nevertheless, advances in the diagnosis and treatment of BC have considerably reduced the mortality rates over the past few years. Typically, BC diagnosis is based on imaging (mammography and ultrasound) and biopsy, and approved circulating biomarkers for diagnosing BC are currently unavailable [2]. In addition, intense debate exists concerning the usefulness of potential biomarkers for monitoring patients during follow-up and evaluating the response of patients with advanced BC to therapy [3,4,5]. Therefore, an unmet need is to search for reliable novel non-invasive biomarkers capable of diagnosing BC. Considerable research efforts to identify circulating biomarkers with potential clinical utility for diagnostic purposes are ongoing. However, a major barrier to assigning the role of early diagnostic biomarker to a circulating molecule relates to the intrinsic and significant inter-patient heterogeneity of this disease [6].

In this framework, microRNAs (miRNAs), a large family of small (20–22 nucleotides) non-coding RNAs, may be useful as diagnostic and prognostic biomarkers [7]. By binding to specific sites within the 3′-UTR (three prime untranslated region), miRNAs decrease the expression of various mRNAs at the post-transcriptional level, leading to either mRNA degradation or the inhibition of protein translation [8]. Based on accumulating evidence, miRNAs play important roles in various critical biological processes, and their deregulation is associated with a broad spectrum of diseases. In particular, miRNAs function as oncogenes or tumor suppressor genes and coordinate multiple cellular processes related to cancer development and progression [9], suggesting that the altered expression of miRNAs not only represents a key pathophysiological component associated with tumor development but also a useful factor for detecting cancer. In support of this hypothesis, miRNAs are released from cells into the extracellular environment in response to various cellular physiological events (apoptosis, necrosis, and active secretion) and are particularly stable in biological fluids [10]. These two characteristics have enhanced the attractiveness of circulating miRNAs as suitable biomarkers to perform a non-invasive and early evaluation of the presence of tumors through a simple blood draw.

In the last two decades, various studies have been performed to identify aberrantly expressed circulating miRNAs for diagnosing BC. Since the first report was published in 2009, in which Zhu and colleagues hypothesized that circulating miRNAs are present and differentially expressed in the serum of patients with BC compared to that of controls [11], extensive data have confirmed profound alterations in the expression of miRNAs in patients with BC, and many circulating miRNAs have emerged as promising diagnostic biomarkers [12]. However, despite the extensive research efforts, circulating miRNAs have not been applied in clinical practice. Many questions related to the significance of documented qualitative and quantitative alterations in miRNA levels remain unsolved, and many challenges in translating the potential of these molecules as novel diagnostic biomarkers for BC into clinical practice persist.

Several studies have critically reviewed the diagnostic or prognostic validity of circulating miRNAs in several human oncological diseases [13,14,15] and discussed the wide range of pre-analytical (sample collection, sample type, sample processing, and nucleic acid extraction), analytical (technological platform for miRNA discovery and validation), and post-analytical (normalization and data processing) pitfalls that contribute to the non-reproducibility of circulating miRNA results. Although these technical approaches are important, they affect the final result, regardless of the pathologies. Our manuscript aims to provide an in-depth analysis of two pivotal and essential requirements for ensuring the clinical validity of identified miRNAs for BC diagnosis through a careful evaluation of the BC literature: i) Specificity and ii) consistent expression between the blood and tissue. In addition, we provide an overview of current clinical trials on circulating miRNAs in patients with BC. In conclusion, we highlight future challenges that must be resolved to address the meaningful clinical use of circulating miRNAs as a novel diagnostic tool.

## 2. The Specificity of Identified Circulating miRNAs for Diagnosing Breast Cancer

**Figure 1:**
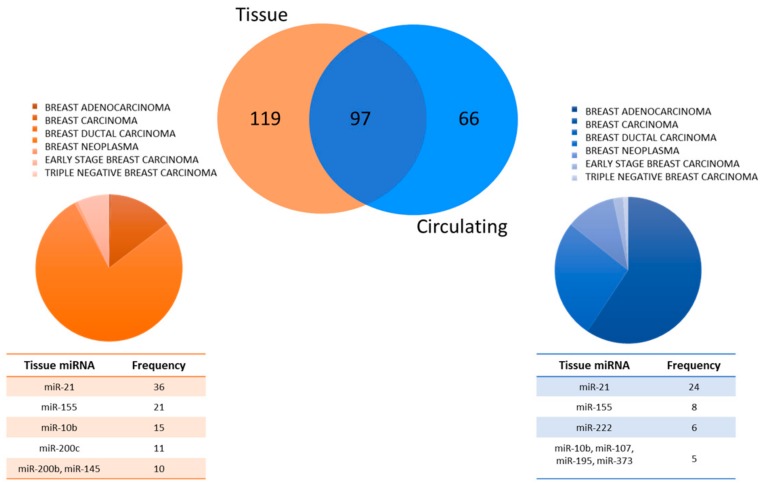
Overview of data for breast cancer (BC)-associated microRNAs (miRNAs) found in Human miRNA Disease Database (HMDD) v3.0. The Venn diagram depicts the fractions of unique and shared circulating and tissue BC-associated miRNAs; pie charts show disease categories of BC for each of the two fractions of miRNAs. Tables list more frequently recurring BC-associated miRNAs observed among all tissue miRNAs and circulating miRNAs.

A broader and more comprehensive literature review was performed using the open web-based resource Human miRNA Disease Database (HMDD v3.0) to investigate the claim that circulating miRNAs are truly specific for BC [16]. This database was built in 2007 to satisfy the increasing demand of scientists investigating the roles of tissue and circulating miRNAs in diseases. The HMDD database collects miRNAs that are associated with the development and progression of a broad range of diseases. All data are experimentally supported and manually curated. The most recent version (HMDD v3.0) collects 32,281 experimentally supported miRNA-disease association entries, including 1102 miRNA genes and 850 diseases from 17,412 papers (last updated on January 3, 2019). Among all miRNAs included in the database, we selected those associated with the spectrum of breast tumors according to the following disease categories: “Breast adenocarcinoma, breast carcinoma, breast ductal carcinoma, breast neoplasm, early stage BC, hereditary BC, and triple negative breast carcinoma”. We identified 281 different BC-associated miRNAs: 216 were reported to be deregulated in BC tissues in 182 papers, 163 were reported as deregulated circulating miRNAs in 91 publications, and 97 miRNAs were shared between these two categories (Figure 1). The most frequently recurring BC tissue-associated miRNAs were miR-21, miR-155, miR-10b, miR-200c, miR-200b, and miR-145, while the most frequently observed circulating BC-associated miRNAs were miR- 21, miR-155, miR-222, miR-10b, miR-107, miR-195, and miR-373 (Figure 1).

**Figure 2:**
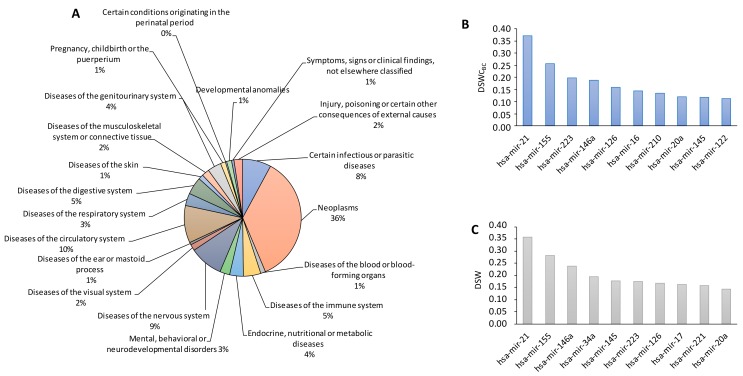
Overview of disease specificity of miRNAs. (**A**) Pie chart depicting the overlap of BC-associated circulating miRNAs with other diseases classified according to international disease classifications (WHO). (**B**) The top 10 BC-associated circulating miRNAs with the highest DSWcBC. (**C**) The top 10 miRNAs with the highest disease spectrum widths (DSWs) in overall HMDD v3.0.

For each of these molecules, we queried HMDD v3.0 and collected and listed all associated human pathologies in a single table (Table S1) to assess the specificity of the identified circulating miRNAs (163). Globally, 88% of the 163 BC-associated circulating miRNAs were deregulated in 291 different additional human diseases (Table S1, columns D to KI). For practicality, we grouped the sorted 291 human diseases into macro-categories according to the World Health Organization (WHO) International Disease Classification 2018 [17]. The identified 291 diseases were classified into 20 of the 24 macro-categories reported in the aforementioned classification (for more details, see Table S1, columns KK to LG), and most of the diseases belonged to the neoplastic category (Figure 2A).

Among the 163 circulating miRNAs, miR-21 was the most non-specific (shared by 108/291 different diseases, of which approximately 54% were neoplastic diseases). Moreover, miR-155 (75/291 pathologies, of which approximately 43% were neoplastic diseases), miRNA-223 (58/291 pathologies, of which approximately 48% were neoplastic diseases), and miR-16 (42/291, of which 50% were neoplastic diseases) exhibited a broad unspecific origin. In HMDD v3.0, the engagement of a miRNA in human diseases is assessed by the “disease spectrum width” (DSW), which is calculated as the ratio between the number of diseases associated with miRNA and the total number of diseases [18]: A higher score indicates a greater number of pathologies in which the miRNA was identified. According to the same criteria, we recalculated the DSW score considering only the BC-associated circulating miRNAs (163) and the number of pathologies associated with these molecules (291), which is referred to as “disease spectrum width circulating breast cancer” (DSWcBC) (Table S1, column KL). After comparing the DSW and DSWcBC scores (Figure 2B,C), we verified that miR-21, miR-155, miR-223, miR-146a, miR-126, and miR-145 were the most non-specific BC-associated miRNAs in both analyses. These findings raised questions about the usefulness of these molecules because of a lack of specificity, although many studies have proposed their use as diagnostic biomarkers for BC. Would the identification of a circulating miRNA panel bypass the lack of specificity for diagnosing BC? Thus, we examined whether circulating miRNA panels emerged from the literature for diagnosing BC. We selected studies that proposed to screen three or more circulating miRNAs for diagnosing BC. We exploited HDDM and PubMed to perform this analysis. We selected 31 studies and 31 panels with 128 circulating miRNAs [19,20,21,22,23,24,25,26,27,28,29,30,31,32,33,34,35,36,37,38,39,40,41,42,43,44,45,46,47,48] (Table S2). 

As summarized in Table 1, 12 circulating miRNAs were shared between three or more panels, but their overlap across the selected 31 panels was poor. This analysis underlined the lack of consistency between the circulating miRNA panels identified by different research groups, making the definition of a reliable circulating miRNA panel for diagnosing BC difficult.

Among all 163 BC-associated circulating miRNAs, only 12% (20 miRNAs) were exclusively associated with breast tumors (miR-190, miR-488, miR-520h, miR-526a-1, miR-526a-2, miR-567, miR-651, miR-801, miR-922, miR-1323, miR-1469, miR-1471, miR-2355, miR-3130-1, miR-3130-2, miR-3186, miR-4257, miR-4417, miR-4728-3p, and miR-6861). A more in-depth analysis of these miRNAs revealed that most of these molecules were proposed as prognostic and predictive blood-associated biomarkers: miR-190 (plasma [48,49]), pre-miR-488 (plasma and serum [50]), and miR-4728-3p (plasma [51]). In addition, miR-520h (human BC cell lines [52]), miR-567 (in silico [53]), miR-4417 (tissue [54]), and miR-651-5p (in silico [55]), which are listed in the HMDD v3.0 database as circulating miRNAs, were assessed in tissues and cell lines but not in blood samples. The only circulating miRNAs that were specifically associated with the early detection of BC were downregulated miR-526 [26], miR-1469 [26], miR-1471 [26], miR-2355 [26], miR-3130-1 [26], miR-3130-2 [26], and miR-3186 [26] and upregulated miR-922 [26], miR-1323 [26], miR-4257 [26], miR-801 [31], and miR-6861 [42]. Although this extracted list provided specific circulating miRNAs that correlated with the BC diagnosis, each of these molecules was reported in only one paper, making their validation for diagnostic purposes improper and premature.

## 3. Consistent Expression of Identified Circulating miRNA in Blood and Tumor Tissues

The most obvious hypothesis proposed by researchers aiming to assign a function of tumor markers to circulating miRNAs is that these molecules are generated from and secreted by the tumor cells into the bloodstream. If this hypothesis is correct, then any circulating miRNAs displaying altered levels in the blood of oncology patients should also be altered in the tumor tissue and show the same trend (upregulated or downregulated). This consistent expression pattern is helpful in validating a diagnostic signature, as the identified circulating miRNAs should be unmistakably related to the tumor. Based on this hypothesis, we examined how many BC-associated miRNAs were screened in both the bloodstream and tissue in the same paper. A literature review was conducted in March 2019 using the online database PubMed. The initial search criteria used the sentence “circulating miRNA breast cancer” and yielded approximately 260 individual publications. A more stringent selection was performed by excluding published studies focused on circulating exosomes, the prediction of therapeutic responses, predictions of metastasis, in vitro experiments with cell lines, methods, drug toxicity, reviews, and meta-analyses, decreasing the number to approximately 80 publications. Of these studies, only 5% analyzed the expression levels of the selected miRNAs in both the tumor tissues and blood samples (serum or plasma) of patients with BC. A second search was performed using the sentence “miRNA blood and tissue and breast cancer”, yielding approximately 141 publications. Using the same stringent selection criteria, only 10.5% of the studies performed in the same paper reported a BC miRNA signature in cancer tissues and blood samples, either through direct experimentation or in silico studies.

After combining the two searches, we selected 19 studies (8.6%) that validated a BC miRNA signature both in the bloodstream and in tissue (Table 2). We grouped these studies into biological samples: six used plasma, 11 used serum, one used whole blood, and one used both plasma and serum. 

In silico approaches were used in three studies as a replacement for direct tissue experiments [64,65,66]. Qattan and colleagues identified a miRNA profile in plasma and verified the expression of these molecules in the tissue. These authors used a miScript miRNA PCR array and found that 17 circulating miRNAs were differentially expressed in the plasma samples from patients with BC compared with those from healthy donors, and the miR-let-7 family and miR-195 were upregulated and significantly correlated with luminal and triple-negative BC (TNBC) subtypes [65]. On the other hand, the tissue expression of these two miRNAs in The Cancer Genome Atlas (TCGA) database was discordant because both miRNAs were downregulated. Yu et al. used an opposite study design; these authors first selected BC miRNA candidates from the TCGA database and then validated these miRNAs in the serum samples from patients with BC and controls [64]. The authors found that a circulating miRNA signature including miR-21-3p, miR-21-5p, and miR-99a-5p, which were upregulated in both the tissue and blood of patients with BC, showed the highest BC diagnostic accuracy. Moreover, Li and colleagues analyzed the miRNA array profiles of 1299 patients with BC collected from the Molecular Taxonomy of Breast Cancer International Consortium (METABRIC) database and analyzed altered miRNA expression between TNBC and non-TNBC subtypes [66]. These authors identified miR-105 and miR-93-3p as diagnostic biomarkers for TNBC, as both molecules were upregulated in the tissue and plasma. Although two of these studies focused on circulating miRNA signatures for diagnosing TNBC, the molecules identified as potential diagnostic biomarkers were completely different.

Among all miRNAs included in Table 2, miR-21 was the most commonly validated and proposed as a potential diagnostic biomarker for BC either alone [69] or in combination with other circulating miRNAs [43,56,61,64,70]. Regardless of the biological sample (plasma or serum), miR-21 was always upregulated both in the bloodstream and tissue. Based on these results, miR-21 represents an appealing choice as a potential BC diagnostic biomarker, but unfortunately, this molecule lacks specificity because it is deregulated in many different human pathologies, as reported in the previous section (Table S1). Similar to miR-21, miR-155 was univocally upregulated both in the tissue and the bloodstream [56,58], but it also lacks specificity (Table S1). Another identified BC-associated miRNA was miR-195, which, in contrast to miR-21 and miR-155, displayed a different expression pattern in the tissue and bloodstream. In fact, Qattan et al. [65] and Cecene et al. [62] reported the upregulation of miR-195 in the bloodstream (plasma and serum) of patients with cancer, but this miRNA was downregulated in the tumor tissue. According to Thakur and colleagues, miR-195 is downregulated in both the tissue and blood (serum) samples from patients with BC [61], and Heneghan and colleagues observed its upregulation in both tissues and blood (serum) samples [68]. Other miRNAs that were reported to be correlated with BC in these two studies were miR-145 and miR-210, but these results appeared somewhat confusing. In fact, miR-210 was upregulated in the bloodstream (plasma) and downregulated in tissue [43] or upregulated in both samples [61]. Similarly, miR-145 was upregulated [33] or downregulated [61] in the bloodstream and tissue. All other miRNAs reported in Table 2 have been identified as circulating diagnostic biomarkers in only one study [27,32,37,59,60,63,66,67]. With the exception of miR-21 and miR-155, the other selected miRNAs showed a different expression patterns in the bloodstream and in tissue. What factors contributed to this discrepancy? Certainly, a major barrier to assigning the role of early diagnostic biomarker to a circulating molecule relates not only to the intrinsic and significant inter-patient heterogeneity but also to the heterogeneity of the pathology itself, since each of the four tumor subtypes of BC might be identified by specific circulating markers. Thus, this heterogeneity adds confusion to an already complex situation in itself. In addition, the lack of a standardized protocol, which is carefully addressed in many studies [2,71,72,73], and the differences in study design potentially affect the results. In fact, some studies selected miRNA signatures from tissue and validated these results in the bloodstream, whereas other studies validated miRNA signatures in the bloodstream and then verified the results in tumor tissues. Additionally, some studies used high-throughput molecular biology technologies with complex computing and math techniques (bioinformatics analysis), whereas others analyzed a few miRNAs selected from the literature, some of which only analyzed one molecule, to validate their use for diagnostic purposes. How does the sample size affect the results? Unfortunately, only a few studies analyzed a cohort that satisfied the statistical requirements for both the discovery cohort and the validation cohort [32,33,40]. Although these studies were well designed and performed using high-throughput technology, their results were different. In one study, the combination of miR-145 and miR-451 successfully discriminated patients with BC from healthy controls [33]. According to Chan et al., the best BC circulating miRNA signature included the upregulation of miR-15b, miR-16, miR-17, miR-25, miR-93, miR-107, and miR-185 and the downregulation of miR-199a-5p [32]. However, Matamala and colleagues proposed the utility of plasma miR-505-5p, miR-125b-5p, miR-21-5p, and miR-96-5p as non-invasive BC diagnostic biomarkers [40]. Have too few studies simultaneously analyzed the miRNA signature in the bloodstream and tissue? We grouped these studies by miRNAs and assessed which molecules were similarly deregulated in the bloodstream and tissue, even if they were studied in different papers, to increase the number of studies. For this analysis, we used HMDD v3.0 and selected all miRNAs that were i) identified in the last five published studies, ii) displayed concordant expression in the blood and tissue, and iii) exhibited partially concordant expression (Table S3). We excluded miRNAs with an unequivocally different expression pattern in the bloodstream and tissue. The selected miRNAs were grouped in Table 3 to help the reader. 

This table includes a list of miRNAs whose expression in tissue and in the bloodstream was concordant (Table 3A; miR-155, miR-373, miR-20a/b, and miR-181b) and partially concordant (Table 3B: miR-21, miR-10b, miR-200c, miR-145, miR-222, miR-200b, miR-195, miR-210, miR-182, and miR-19a). Among the miRNAs listed in Table 3A, miR-155 is more appealing because it has been cited in 16 studies. Interestingly, the concordant upregulation of miR-155, even if it was extracted from separate studies, was also observed in Table 2, which lists the miRNAs analyzed in the bloodstream and tissue in the same study [56,58]. Similarly, the concordant upregulation of miR-20 was also reported in Table 2 [67]. Thus, the miRNAs that were most frequently observed in BC studies displayed higher variability in expression between the blood and tissue (Table 3B). Nevertheless, for miR-21 and miR-145, a consistent expression pattern was observed in the circulation and tissue in 90% of the studies (miR-21: Upregulated and miR-145: Downregulated), while approximately 75% and 80% concordance was observed for miR-222 (upregulated) and miR-195 (downregulated), respectively. For the remaining miRNAs, the results were confusing. The trends of miR-21 and miR-222 expression reported in Table 3 matched very well with the pattern reported in Table 2, while the trends of miR-145 and miR-195 expression were partially consistent.

Taken together, among all BC-associated circulating miRNAs analyzed in this section, the most attractive candidates for diagnostic purposes were a panel including miR-155, miR-21, miR-195, and miR-145. Nevertheless, with the exception of miR-195, we were unable to exclude the possibility that miR-155, miR-21, and miR-145 are the most non-specific BC-associated miRNAs identified in the previous section.

## 4. Clinical Trials Including Breast Cancer-Associated miRNAs

We queried ClinicalTrials.gov, a database of privately and publicly funded clinical studies conducted worldwide, to provide a snapshot of recent developments in the assessment of circulating miRNAs as diagnostic biomarkers for BC and their clinical translation. We searched all studies for the terms “Breast cancer” in the field “disease/condition” and “miRNA” in the field “other terms”. Approximately 39 clinical trials are examining miRNAs, of which 24 were interventional and 15 were observational studies. Most of these studies aimed to evaluate the effect of miRNAs on predicting the response to therapeutic treatments. After restricting the search to only circulating miRNAs (terms used for the search: “Breast cancer” and “circulating miRNA”), we only identified seven studies, which are summarized in Table 4. 

Some studies aim to identify a panel of circulating miRNAs to discriminate patients with BC who respond to neoadjuvant and adjuvant chemotherapy (NCT01722851, NCT01612871, and NCT03779022) or who develop resistance to neoadjuvant chemotherapy (NCT03255486). The ANDROMEDA Study (NCT02618538) aims to create possible customized screening paths, including circulating miRNAs, through a combined analysis of the main risk factors for BC. Two other projects aim to assess the impact of intervention models of lifestyle changes (diet and physical activity) on healthy subjects (NCT03118882) or affected patients during follow-up (NTC03528473). Three of the selected studies have completed patient enrolment, while others are still recruiting patients. None of these trials have reported the results. An analysis of the time frame revealed that the first clinical trial on circulating miRNAs in patients with BC started in 2010, the total number of trials including patients with BC from 2010 to the present is approximately 5500, with less than 0.2% examining circulating miRNAs, and none focusing on evaluating the ability of these molecules to diagnose BC. These data led us to speculate that their application in diagnostics is still premature.

## 5. Conclusions

Circulating miRNAs represent an appealing class of molecules for non-invasive diagnostics, but despite extensive research efforts, their translation into clinical practice appears to be a future goal. This review analyses the literature investigating the role of circulating miRNAs as diagnostic biomarkers for BC. Nevertheless, we deliberately avoided commenting on the effect of the lack of standard protocols in the pre-analytical and analytical phases on the final result, as the technical aspects that are responsible for the non-reproducibility of the data (biological fluids, diurnal variations, fasting state, needle gauge, contamination, extraction method, hemolysis, normalization, and the choice of detection platform) have been extensively discussed in previous reviews [13,14,15,74,75,76,77]. Most of these reviews address these issues by examining several cancers, suggesting that the technical aspect is independent of the pathology and standardized procedures for sample choice, preparation, and processing are clearly needed. Here, we examined the literature to specifically investigate two additional fields that are linked to the pathology and are required for the validation of circulating miRNAs for diagnostic purposes in BC: i) Specificity and ii) consistent expression between the blood and tissue. Therefore, we extracted information from both the HMDD v3.0 database and individual papers. An in-depth analysis of the data suggested that circulating miRNAs were identified as potential diagnostic biomarkers for BC lacked specificity and often showed discordant expression in the blood and tissue of patients affected with cancer. The final processing of our in-depth analysis revealed that a circulating miRNA signature for BC diagnosis is currently unavailable because the most frequently studied circulating miRNA, BC-associated miRNAs (miR-155, miR-21, miR-195, and miR-145), except for miR-195, are the most non-specific BC-associated miRNAs, although some of these miRNAs were able to discriminate patients with BC from patients with benign breast lesions (BBLs) (miR-21 [69,78]; miR-155 [79,80]; miR-145 and miR-195 [61]). Factors triggering neoplastic transformation may differ among tumors, but the common denominator for all neoplastic diseases is uncontrolled cell growth and the activation of inflammatory processes. If these circulating miRNAs correlate with different oncological diseases, then they likely participate in signals that are common in different cancers, and these common molecules are easily detected in the bloodstream because they exhibit the highest variation in expression between healthy and affected subjects. Consequently, their release in the bloodstream might mask cancer-specific molecules with a fold change < 2 but are subsequently discarded by researchers. In addition, the breast tissue is not the only contributor to circulating miRNAs. The immune system and its cellular components, particularly peripheral blood mononuclear cells (PBMCs), function as the first line of defense against cancer. Differential expression of various molecules in PBMCs have been identified in oncology patients, confirming that the assessment of miRNA and gene expression in PBMCs may provide a new diagnostic approach for the early detection of cancer [57,81,82,83,84]. Is it a plausible hypothesis that miRNAs released into the bloodstream of affected patients originate not only from tumor tissues but also from PBMCs? This hypothesis would make the interpretation of the data even more complicated. We propose that a possible strategy is the simultaneous analysis of miRNA expression in tissues and PBMCs from patients with BC to select a signature and verify it in the bloodstream. Again, in the last few years, different studies focused on determining the correlations and integration among molecular and imaging biomarkers for diagnostic and prognostic purposes. The simultaneous use of these apparently different tools to answer a clinical question is called radiogenomics and is aimed at correlating cancer imaging and gene features [85]. A blood-based biomarker profile combined with imaging findings might increase both the sensitivity and specificity of the diagnosis. To date, circulating miRNAs have been combined with mammography parameters [86,87], indicating that this association improves the accuracy of identifying malignant breast lesions. Another possible strategy for validating circulating miRNAs is their combination with validated protein markers for breast cancer [88,89]. Additionally, absolute quantification and defined optimal cut-off values for miRNAs are required for the development of clinically applicable tools. Large prospective multicenter studies and analyses of independent patient cohorts with different tumor stages and different subtypes may provide useful data for identifying diagnostic circulating miRNA panels.

## Figures and Tables

**Table 1:** Number of BC-associated circulating miRNA panels and their overlap.

Total Number of Panels	Recurring Circulating miRNAs	Circulating miRNA Frequency in Panels
31	miR-10b	3
	miR-16	5
	miR-21	8
	miR-24	3
	miR-29a	4
	miR-107	3
	miR-145	3
	miR-155	7
	miR-195	3
	miR-200c	3
	miR-222	4
	miR-373	3

**Table 2:** List of miRNAs analysed in the bloodstream and in tissue in the same study.

Sample	Bloodstream Expression	Tissue Expression	Concordance	Patients (N)	Method	Reference
Serum	↑ miR-21, miR-106a and miR-155; ↓ miR-126, miR-199a and miR-335	↑ miR-21, miR-106a and miR-155; ↓ miR-126, miR-199a and miR-335	Yes	68 BC tissues and adjacent non-tumour tissues and matching serum samples and 40 normal control sera	RT-qPCR	[56]
	↑ miR-222	↑ miR-222	Yes	Screening step: 5 BC tissues and adjacent normal tissues and 13 BC serum and 10 healthy control serum. Validation step: 20 BC tissues and adjacent normal tissues and 50 BC serum and 50 healthy controls serum and 20 ovarian cancer serum and 20 benign breast cancer serum	SOLiD sequencing/RT-qPCR	[27]
	↑ miR-15b, miR-16, miR-17, miR-25, miR-93, miR-107 and miR-185; ↓ miR-199a-5p	↑ miR-15b, miR-16, miR-17, miR-25, miR-93, miR-107 and miR-185; ↓ miR-199a-5p	Yes	Markers discovery: 32 BC tissues and adjacent normal tissues and 32 BC serum and 22 healthy control serum. Validation stage: 132 BC serum and 101 healthy control serum.	LNA RT-PCR human miRNA panels (Exiqon)/RT-qPCR	[32]
	↑ miR-21; ↓ miR-92a	↑ miR-21; ↓ miR-92a	Yes	48 BC tissue and 100 BC serum and 20 healthy control serum.	RT-qPCR	[57]
	↓ miR-205; ↑ miR-155	↓ miR-205; ↑ miR-155	Yes	4 BC tissues and adjacent normal tissues and 20 BC serum and 10 healthy donors serum.	Microarray Chip/RT-qPCR	[58]
	↓ let-7c	↓ let-7c	Yes	90 BC serum and 64 healthy control serum.	RT-qPCR	[59]
	↑ miR-182	↑ miR-182	Yes	3 BC tissues and adjacent normal tissues; 46 BC serum and 58 healthy control serum.	RT-qPCR	[60]
	↑ miR-21, miR-221, miR-210, let-7a; ↓ miR-195, miR-145	↑ miR-21, miR-221, miR-210 and Let-7a; ↓ miR-195, miR-145	Yes	85 BC tissues and adjacent normal tissues and 85 BC serum and 85 healthy donors serum; 15 benign breast tissues and adjacent normal tissues and 15 benign breast tissues serum and 15 healthy donors serum.	RT-qPCR	[61]
	↑ miR-195	↓ miR-195	No	96 BC tissues and normal tissues; 48 pre-operative BC serum and 48 post-operative BC serum and 12 heathy control serum.	RT-qPCR	[62]
	↑ miR-139	↑ miR-139	Yes	74 BC tissues and 18 paired serum and tissue samples of BC and 10 healthy donor serum.	RT-qPCR	[63]
	↑ miR-21-5p and miR-21-3p;↓ miR-99a-5p	↑ miR-21-5p and miR-21-3p; ↓miR-99a-5p	Yes	In silico (TGCA): 409 BC tissue and 87 healthy controls tissues. Samples 113 BC serum and 47 healthy controls serum.	RT-qPCR	[64]
Plasma	↓ miR-181a, miR-652, miR-29a and miR-223	↑ miR-181a and miR-652	No	11 BC tissues and adjacent normal tissue and 54 Luminal A-like breast cancer plasma and 56 healthy control plasma.	TaqMan human miRNA arrays/RT-qPCR	[37]
	↑ miR-505-5p, miR-125b-5p, miR-21-5p, miR-96-5p	↑ miR-21-5p, miR-96-5p; ↓ miR-505-5p, miR-125b-5p	Partially	Markers discovery: 122 BC tumours and 11 healthy breast tissue. Discovery cohort: 83 BC plasma and 26 healthy control plasma. Validation cohort: 114 BC plasma and 116 healthy control plasma.	(LNA)-based miRNA microarrays	[40]
	↑ miR-21, miR-146a, and miR-210	↑ miR-21 and miR-146a; ↓ miR-200c and miR-210	Partially	89 BC tissues; 30 not BC tissues; 55 BC plasma and 20 healthy donors plasma.	RT-qPCR	[43]
	↑ miR-145 and miR-451	↑ miR-145 and miR-451	Yes	Markers discovery: 5 BC tissue and adjacent normal tissue and 5 BC plasma and 5 normal control plasma. Marker validation: 170 BC plasma and 100 normal control plasma. Blind validation: 70 BC plasma and 50 normal control plasma.	TaqMan Array Human MicroRNA Panels A and B/RT-qPCR	[33]
	↑ let-7 miRNA and miR-195	↓ let-7 miRNA and miR-195	No	In silico (TGCA): 120 luminal samples and 38 Triple Negative samples and 87 control. Samples: 57 luminal BC plasma and 36 TNBC plasma and 34 healthy control.	miScript miRNA PCR Array /RT-qPCR	[65]
	↑ miR-105 and miR-93-3p	↑ miR-105 and miR-93-3p	Yes	In silico (Metabric): 204 TNBC and 1095 non-TNBC from Metabric database; 94 TNBC and 79 non-TNBC from GEO dataset. Samples: 13 TNBC normal and tumour tissue pairs and 12 non-TNBC N-T pairs and 130 plasma samples (12 healthy controls, 74 TNBC and 44 non-TNBC).	RT-qPCR	[66]
Plasma and Serum	↑ miR-106a-5p and miR-20b-5p	↑ miR-106a-5p and miR-20b-5p	Yes	200 BC plasma and 200 plasma controls and 204 BC serum and 202 serum control; 32 paired breast tissues.	RT-qPCR	[67]
Whole blood	↑ miR-195	↑ miR-195	Yes	65 BC tissues and adjacent normal tissues and 83 BC whole blood and plasma and serum and 44 healthy control whole blood and plasma and serum.	RT-qPCR	[68]

**Table 3:** List of miRNAs analysed in the bloodstream and in tissue in separate studies.

**A.** **Concordant**					
**miRNA**	**Total Studies (n°)**	**Circulating Expression**		Tissue Expression	
		**Up**	Down	Up	Down
mir-155	16	4	-	12	-
mir-373	6	3	-	3	-
mir-20a-b	6	4	-	2	-
mir-181b	5	1	-	4	-
**B.** **Partially Concordant**					
miRNA	Total Studies (n°)	Circulating Expression		Tissue Expression	
		Up	Down	Up	Down
mir-21	38	10	1	25	2
mir-10b	12	2	1	5	4
mir-200c	10	1	1	3	5
mir-145	11	-	3	1	7
mir-222	8	3	2	3	-
mir-200b	6	1	-	2	3
mir-195	5	1	2	-	2
mir-210	6	1	1	3	1
mir-182	8	2	2	2	2
mir-19a	5	1	1	1	2

**Table 4:** Selected list of clinical trials involving circulating miRNAs.

Identifier	Status	Study Type	Study Start Date	Title	Interventions
NCT01612871	Completed	Interventional	2012	Circulating miRNAs as Biomarkers of Hormone Sensitivity in Breast Cancer	Drugs: -Tamoxifen -Letrozole -Anastrozole -Exemestane
NCT01722851	Recruiting	Observational	2011	Circulating miRNAs. ICORG 10-11, V2	-none
NCT03779022	Recruiting	Observational	2015	miRNA and Relevant Biomarkers of BC Patients Undergoing Neoadjuvant Treatment	Genetic: -microRNA
NCT02618538	Enrolling by invitation	Observational	2015	The ANDROMEDA Study. Predictive Value of Combined Criteria to Tailor Breast Cancer Screening	-none
NCT03255486	Completed	Interventional	2013	Identification and Evaluation of Biomarkers of Resistance to Neoadjuvant Chemotherapy (IDEA SEIN)	Biological: -Blood sample
NCT03528473	Recruiting	Interventional	2019	Adapted Physical Activity (APA) in a Breast Cancer Population	Behavioural: -Exercise
NCT03118882	Completed	Interventional	2010	STI.VI. Study: How to Improve Lifestyles in Screening Contexts	Behavioural: -Diet -Physiological activity

## Supplementary Materials

The following materials are available online at https://www.mdpi.com/2072-6694/11/7/901/s1, Table S1: BC-associated circulating miRNAs, Table S2: Circulating miRNA panels, Table S3: miRNA expression patterns in the blood and tissue.
